# Genome-Wide Detection of Copy Number Variations and Their Association With Distinct Phenotypes in the World’s Sheep

**DOI:** 10.3389/fgene.2021.670582

**Published:** 2021-05-20

**Authors:** Hosein Salehian-Dehkordi, Ya-Xi Xu, Song-Song Xu, Xin Li, Ling-Yun Luo, Ya-Jing Liu, Dong-Feng Wang, Yin-Hong Cao, Min Shen, Lei Gao, Ze-Hui Chen, Joseph T. Glessner, Johannes A. Lenstra, Ali Esmailizadeh, Meng-Hua Li, Feng-Hua Lv

**Affiliations:** ^1^CAS Key Laboratory of Animal Ecology and Conservation Biology, Institute of Zoology, Chinese Academy of Sciences (CAS), Beijing, China; ^2^College of Life Sciences, University of Chinese Academy of Sciences (UCAS), Beijing, China; ^3^College of Animal Science and Technology, China Agricultural University, Beijing, China; ^4^State Key Laboratory of Sheep Genetic Improvement and Healthy Breeding, Xinjiang Academy of Agricultural and Reclamation Sciences, Shihezi, China; ^5^Center for Applied Genomics, Children’s Hospital of Philadelphia, Philadelphia, PA, United States; ^6^Faculty of Veterinary Medicine, Utrecht University, Utrecht, Netherlands; ^7^Department of Animal Science, Shahid Bahonar University of Kerman, Kerman, Iran

**Keywords:** sheep, GWAS, CNVs, selection, genetic adaptation

## Abstract

Copy number variations (CNVs) are a major source of structural variation in mammalian genomes. Here, we characterized the genome-wide CNV in 2059 sheep from 67 populations all over the world using the Ovine Infinium HD (600K) SNP BeadChip. We tested their associations with distinct phenotypic traits by conducting multiple independent genome-wide tests. In total, we detected 7547 unique CNVs and 18,152 CNV events in 1217 non-redundant CNV regions (CNVRs), covering 245 Mb (∼10%) of the whole sheep genome. We identified seven CNVRs with frequencies correlating to geographical origins and 107 CNVRs overlapping 53 known quantitative trait loci (QTLs). Gene ontology and pathway enrichment analyses of CNV-overlapping genes revealed their common involvement in energy metabolism, endocrine regulation, nervous system development, cell proliferation, immune, and reproduction. For the phenotypic traits, we detected significantly associated (adjusted *P* < 0.05) CNVRs harboring functional candidate genes, such as *SBNO2* for polycerate; *PPP1R11* and *GABBR1* for tail weight; *AKT1* for supernumerary nipple; *CSRP1*, *WNT7B*, *HMX1*, and *FGFR3* for ear size; and *NOS3* and *FILIP1* in Wadi sheep; *SNRPD3*, *KHDRBS2*, and *SDCCAG3* in Hu sheep; *NOS3*, *BMP1*, and *SLC19A1* in Icelandic; *CDK2* in Finnsheep; *MICA* in Romanov; and *REEP4* in Texel sheep for litter size. These CNVs and associated genes are important markers for molecular breeding of sheep and other livestock species.

## Introduction

Identification of genetic variants associated with phenotypic traits and local adaptation in livestock is crucial for better understanding sheep genomic variants and their molecular breeding in the face of climatic change (Goddard and Hayes, 2009). Copy number variations (CNVs) are a source of structural variation and include deletions and duplications with a size ranging from 1 kb to several Mb (Scherer et al., 2007). Previous studies reported that CNVs account for ∼4.8–9.5% of the human genome and are important in both functional and evolutionary perspectives (Zarrei et al., 2015).

Functionally, CNVs are mainly involved in the changes in gene structure, dosage, regulation, and expression (Redon et al., 2006; Zhang et al., 2009; Conrad et al., 2010). With the rapid development of high-throughput technologies, whole genome scans for CNVs have been an efficient strategy for identifying functional genes associated with physiological (e.g., fitness and metabolism) and pathological (e.g., immune response) processes and phenotypic traits (e.g., body size) in livestock (Fowler et al., 2013; Zhu et al., 2016; Liu et al., 2019b; Wang et al., 2019; Xu et al., 2019).

Following domestication, as many as 1400 distinct populations of sheep (*Ovis aries*) have been developed in the world after long-term local adaptation and artificial selection (Scherf and Loftus, 2000). In particular, a few local populations have been characterized to be adapted to extreme environments such as plateau and arid-desert regions (Yang et al., 2016) and with distinct phenotypic traits such as fat tail and large litter size (Xu et al., 2017, 2018). Recently, the availability of the sheep reference genome (oar_v4.0) and genome-wide single-nucleotide polymorphisms (SNPs) has improved our understandings of genomic diversity, population history, selective pressures, and genetic basis underlying distinct phenotypic traits in sheep (Lv et al., 2014; Xu and Li, 2017; Zhao et al., 2017; Li et al., 2020a). However, as a source of phenotypic variations and environmental adaptation, genome-wide CNVs have mainly focused on Chinese sheep breeds (Liu et al., 2013; Ma et al., 2015; Jenkins et al., 2016; Zhu et al., 2016; Ma et al., 2017; Yang et al., 2018; Li et al., 2020a).

Here, we use the Ovine Infinium HD SNP BeadChip for a genome-wide analysis of CNVs on a world-wide panel of breeds. Our main aims are targeted at (i) characterization of CNV variability across the whole genome within and among populations and (ii) identification of CNVs and relevant functional genes associated with distinct phenotypic traits such as polyceraty, tail weight, supernumerary nipple, litter size, and ear size. Our results will provide novel insights into the genetic mechanisms underlying the local environmental adaptation and phenotypic variations.

## Materials and Methods

### Genotypic and Phenotypic Data

**FIGURE 1:**
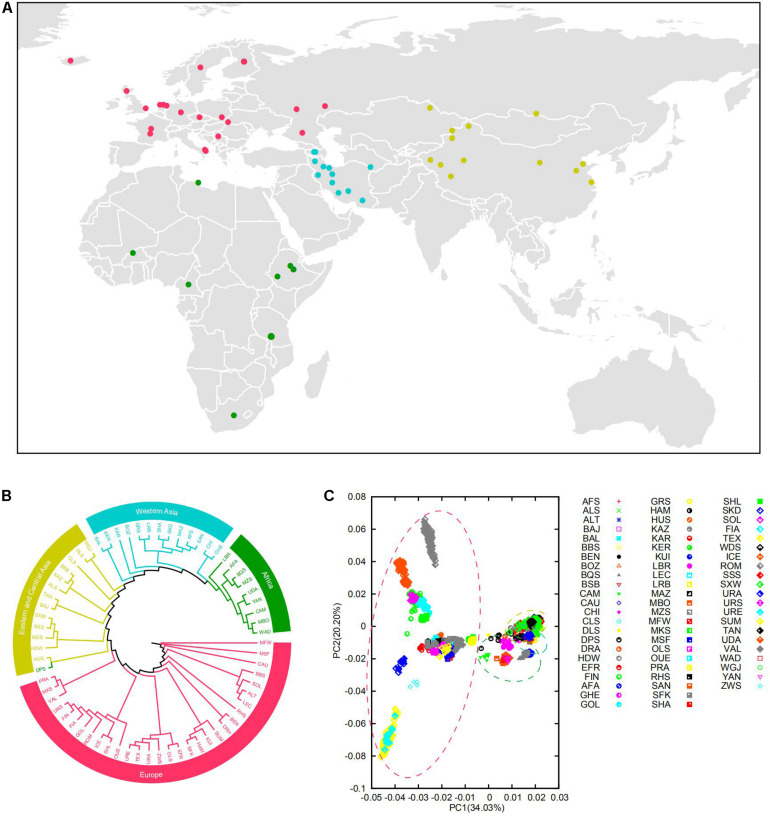
**(A)** Geographic origin of the domestic sheep populations studied. The domestic sheep populations are color coded according to geographical origin. **(B)** Neighbor-joining clustering of the sheep populations based on the Reynolds’ distances. **(C)** Principal component analysis (PCA) of the 67 domestic sheep populations. The circle of yellow, blue, green, and red represent Eastern-Central Asian, Western Asian, African, and European populations, respectively.

We combined the genotypes of Ovine Infinium HD (600K) SNP BeadChip in 2059 individuals from 67 populations all over the world, a large majority of which are from our research group (Kijas et al., 2014; Ren et al., 2016; Xu et al., 2017; Peng et al., 2017; Zhao et al., 2017; Gao et al., 2018; Xu et al., 2018; Rochus et al., 2018; Cao et al., 2020) (Figure 1A and Supplementary Table 1). We updated all the SNP positions in the BeadChip based on the sheep reference assembly Oar_v.4.0^1^.

Phenotypes used for genome-wide association analysis (GWAS) between CNVs and particular traits are extracted from earlier publications: polyceraty for Sishui Fur sheep, tail weight for large- and small-tailed Han sheep, supernumerary nipples for Wadi, litter size for Wadi, Hu, Finnsheep, Icelandic, Romanov, and Texel, and ear size for Duolang sheep (Ren et al., 2016; Xu et al., 2017; Peng et al., 2017; Gao et al., 2018; Xu et al., 2018).

### Quality Control

Quality control was implemented using the PLINK v.1.09 software (Purcell et al., 2007). We removed the SNPs and individuals meeting any of the following criteria: (i) SNPs without chromosomal or physical locations or located on the X- or Y-chromosome; (ii) SNPs with > 0.1 missing data; (iii) individuals with a genotyping rate < 0.9; (iv) SNPs with minor allele frequency (MAF) < 0.05; (v) SNPs with a *P* < 0.00001 for the Fisher’s exact test of Hardy–Weinberg equilibrium (HWE). This resulted in a data set of 1768 individuals genotyped for 514,215 autosomal SNPs.

### Population Differentiation Analysis

Population differentiation was characterized by three different statistical approaches, such as principal component analysis (PCA), neighbor-joining (NJ) tree, and admixture analysis. We used SmartPCA program from the EIGENSOFT v.6.0beta package for the PCA (Patterson et al., 2006). For SNPs, the NJ tree was built based on the pairwise-population Reynolds’s distances (Reynolds et al., 1983) calculated via the Arlequin v.3.5 program (Felsenstein, 1989), while the NJ tree for CNVs was constructed from the Manhattan distances matrix between populations. The genetic distances were calculated from the SNP allele frequencies using the program PEAS v.1.0 (Xu et al., 2010). The NJ tree was visualized by FigTree v.1.4.2^2^. In addition, we assessed the population genetic components based on the maximum-likelihood clustering program Admixture software v.1.23 with *K* values = 2–7 (Alexander et al., 2009).

### Detection of CNVs and CNVRs

We retrieved signal intensity ratios (log R ratio: LRR) and allelic frequencies (B allele frequency: BAF) for each SNP using the Illumina GenomeStudio v1.0 software. These values were used to infer the CNVs for each animal, which were calculated via a hidden Markov model combined with a wave adjustment procedure for genomic waves using the program PennCNV v1.0.5^3^. A subsequent PennCNV quality filtering follows the following criteria: (i) the sample must have a standard deviation of LRR < 0.3, a BAF drift < 0.01, and a waviness factor < 0.05 and (ii) the CNV must contain 10 or more consecutive SNPs. We obtained CNV regions (CNVRs) based on proximity (1 Mb) and similar significance (power of 10 *P* value, Fisher’s exact test) of neighboring SNPs (Glessner et al., 2013). We designated the genotype status of these CNVR as loss (deletions), gain (duplications), or both (deletion and duplication).

We constructed the CNV and CNVR maps for the four groups of populations (see *Results* below). The overlap of CNVRs from different groups was calculated by a homemade python script and was shown in a Venn diagram. Among the 709 CNVRs with at least two CNV events, 36 CNVRs were selected with the top 95% highest variance. We plotted the frequency of the CNV events in these CNVRs within each group in a heatmap. The frequencies of CNV events were also used to calculate the Euclidean distances for hierarchical clustering analysis.

### Genome-Wide Association Analysis Between CNVs and Phenotypic Traits

We carried out CNV-trait GWAS for the following five different phenotypes: (i) for polyceraty, 471 CNVs in 40 Sishui Fur sheep (12 rams and 3 ewes with 4 horns; 19 rams and 6 ewes with 2 horns) (Ren et al., 2016); (ii) for tail weight, 560 CNVs in 102 large-tailed Han sheep with and 703 CNVs in 99 short-tailed Han sheep (Xu et al., 2017); (iii) for supernumerary nipples, 735 CNVs in 69 ewes with 4 nipples and 63 ewes with two nipples in Wadi sheep (Peng et al., 2017); (iv) for litter size, 596 CNVs in 115 Wadi sheep [litter size (LS) ≥ 2 for 92 ewes, LS = 1 for 23 ewes, etc.], 561 CNVs in 77 Hu sheep (LS ≥ 2 for 63 ewes, LS = 1 for 14 ewes, etc.), 510 CNVs in 31 Icelandic sheep (LS ≥ 2 for 17 ewes, LS ≤ 1.75 for 14 ewes, etc.), 159 CNVs in 44 Finnsheep (LS ≥ 2.5 for 35 ewes, LS ≤ 2 for 9 ewes, etc.), 371 CNVs in 64 Romanov sheep (LS ≥ 2.5 for 40 ewes, LS ≤ 2 for 24 ewes, etc.), and 147 CNVs in 42 Texel sheep (LS ≥ 1.6 for 28 ewes, LS ≤ 1.33 for 14 ewes, etc.) (Xu et al., 2018); and (v) for ear size, 4031 CNVs in 101 Duolang sheep with large and variable floppy ears (ear size: 102.55–180 cm^2^, Gao et al., 2018).

We used the programs ParseCNV^4^ and PLINK v1.09 in the association analyses, applying the case–control model for the traits of polyceraty, supernumerary nipple, and litter size and the logistic regression model for tail weight and ear size. First, we used the program ParseCNV, which calculated probe-based statistics for CNV occurrence in both the case–control design and the logistic regression model on the basis of the CNV calls using the code “*–includePed argument.*” Next, we calculated the association between CNVs and phenotypic traits using the Fisher’s exact test for the case–control design and the PLINK codes “*–pheno*” and “*–assoc arguments*” for the logistic regression model. We applied the ParseCNV script “*InsertPlinkPvalues.pl*” to merge adjacent SNP-based CNV occurrence p values into CNVRs. Program ParseCNV was run with 10,000 permutations using the code “*–permuteP* < *10,000* >.” After the multiple testing correction, a nominal significance threshold of max(T) adjusted *P* < 0.05 was used to select CNVRs for pathway analysis.

### Gene Annotation

We annotated the gene content in the CNVRs using the sheep reference assembly Oar_v.4.0. We only considered genes that have an overlap with a CNVR spanning at least 10% of the CNV length using the options “*Bedtools-intersect*” and “*-f 0.1-r.*” We performed Gene Ontology (GO) enrichment and Kyoto Encyclopedia of Genes and Genomes (KEGG) pathway analysis using the database for annotation, visualization, and integrated discovery (DAVID)^5^. We only considered the enriched GO terms and KEGG pathways with the *P* < 0.05 after the Bonferroni correction for multiple testing and at least six genes from the input gene list. We compared the detected CNVRs with the sheep quantitative trait locus (QTL) database^6^. Using Bedtools (Liu et al., 2019a), we only considered QTLs with confidence interval < 5 Mb, which have an overlap with a CNVR spanning at least 50% of the CNV length.

### Quantitative PCR Validation

We performed quantitative PCR (qPCR) to validate the accuracy of the inferred CNVs. In addition to 9 randomly selected CNVs (Supplementary Table 2), 13 CNVs associated with phenotypic traits and encompassing functional genes were selected to validate in 103 cases and 22 controls with different copy number alleles (gain, loss, and both gain and loss) (Supplementary Table 3). *DGAT2* was selected as the reference gene for all the qPCR tests (Ma et al., 2017; Li et al., 2020a). Primers were designed using the software Primer v5.0 (Lalitha, 2000) based on the sheep reference assembly Oar_v.4.0 (Supplementary Tables 2,3). In each of the samples examined, we measured the concentration of the genomic DNA using a NANODROP 2000 spectrophotometer (Thermo Scientific, MA, United States). We used samples without loss or gain as the reference samples. Real-time qPCR assays were performed using the GoTaq qPCR Master Mix kit (Promega, Madison, WI, United States) and the following cycling conditions: 95°C for 5 min, followed by 40 cycles of 95°C for 15 s and 60°C for 60 s, and primer extension at 72°C for 30 s. We calculated the relative copy numbers of the target genes in the test samples using the equation of ΔΔ_T_ = (*C*_T_target_ – *C*_T__*_DGAT2_*)_sample_A_ − (*C*_T_control_ – *C*_T__*_DGAT2_*)_sample_B_, where *C*_T_ is the cycle threshold, sample A is the tested individual, and sample B is the reference individual. We measured the standard deviation of the ΔΔ*C*_T_ value, which is the same as the standard deviation of the Δ*C*_T_ value, and can be calculated as s = (s${}_{1}^{2}$ + s${}_{2}^{2}$)^1/2^, where s_1_ is the variance of the target *C*_T_ value, and s_2_ is the variance of the reference *C*_T_ value (Schmittgen and Livak, 2008; Li et al., 2020b). The ΔΔCT results between 1.414 and 2.449 were considered to represent a normal copy number of 2 (D’haene et al., 2010).

## Results

### CNV and CNVR Detection

**FIGURE 2:**
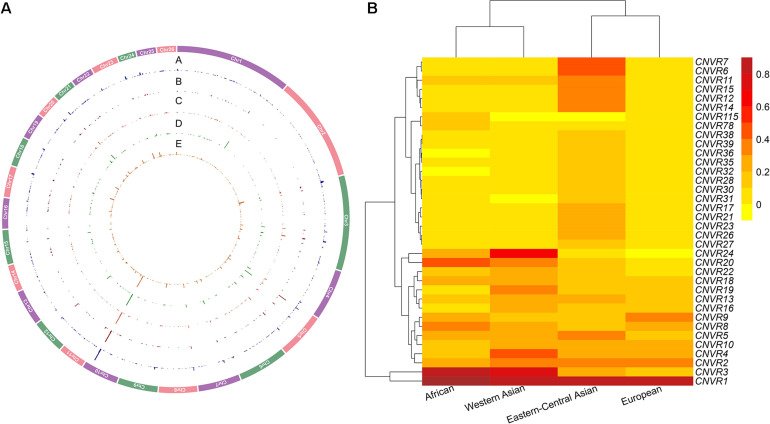
**(A)** Genomic distribution and frequencies of copy number variation regions (CNVRs) on autosomes in the four genetic groups of domestic sheep populations. The tracks from outside to inside **(A–E)** are CNVRs shared across all the five groups: African, Eastern-Central Asian, European, and Western Asian populations. **(B)** CNVR frequency heatmap based on hierarchical clustering analysis for the top 5% of CNVRs (CNV events ≥ 2 CNV) in the four genetic groups of domestic sheep populations: African, Eastern-Central Asian, European, and Western Asian populations. The value is the number of CNVRs per individual (total of CNVRs/sample size based on population size in each CNVR).

**FIGURE 3:**
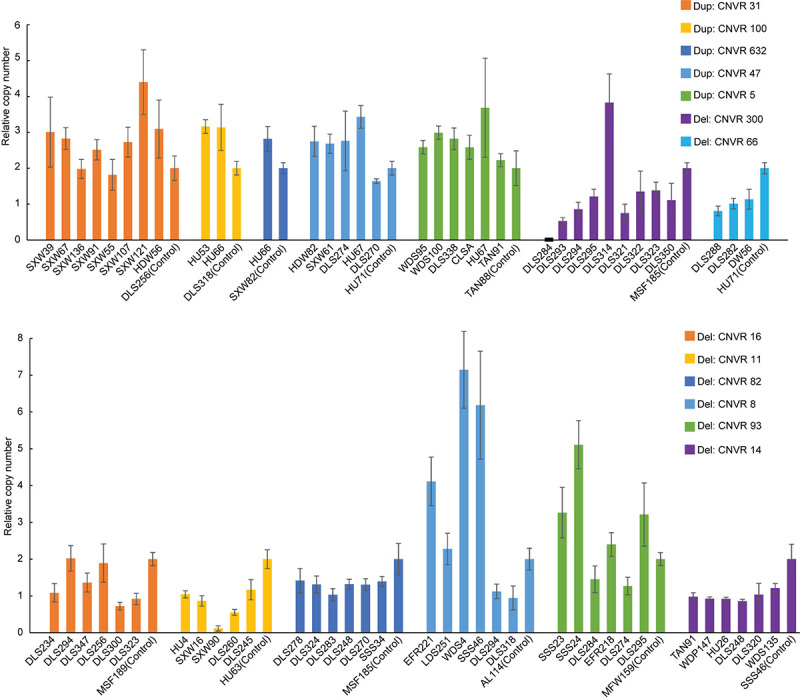
Validation of copy number variations (CNVs) (i.e., five duplications and eight deletions) by quantitative PCR (qPCR). The *x-* and *y*-axes represent sample ID and relative CNVs expression level [2 × 2^–(ΔΔCT±SD)^, *n* = 3].

After quality filtering, we obtained a total of 18,152 CNV events (Table 1 and Supplementary Table 4). On average, 10 CNV events were found for each individual with an average length of 104.8 kb, but standard deviations indicate a considerable variation. We also observe a large spread of CNV coverage per chromosome from 8 to 80% (Figure 2A, Supplementary Table 5). The 18,152 CNV events were merged into 1217 non-redundant CNVRs including 918 losses, 197 gains, and 102 for both losses and gains in the same region, totaling a length of approximately 245 Mb and around 10% of the whole genome (Supplementary Table 6). We obtained a validation rate of ∼85.3% for the nine randomly selected CNVs, which represent different copy number status (gain, loss, and both gain and loss) (Supplementary Figure 1 and Supplementary Table 7). In addition, 13 selected CNVs, which were significantly associated with phenotypic traits by association analysis (see *Results* below), were successfully validated using qPCR, and 78.05% concordant genotypes (22/27 duplications and 42/55 deletions) were confirmed (Figure 3 and Supplementary Table 3).

**TABLE 1:** Summary of copy number variations (CNVs) and CNV regions (CNVRs) identified from the world’s sheep populations.

Groups	CNV										Unique CNVs				Non-redundant CNVR			
	No. Ind.	Count		Gain		Loss		Length			Count	Length			Count	Length		
		Total	Avg.	Total	Avg.	Total	Avg.	Total (Mb)	Avg. (kb)	S.D. (kb)		Total (Mb)	Avg. (kb)	SD (kb)		Total (Mb)	Avg. (kb)	SD (kb)
Eastern-Central Asian	778	10521	13.5	2631	3.4	7890	10.1	1132.7	107.6	101.7	5245	632.7	120.6	131.91	970	159.7	164.6	273.3
Western Asian	175	1595	9.1	670	3.8	925	5.3	153.4	96.2	63.3	819	86.9	106.1	78.4	304	36.12	119.2	116.35
African	120	1119	9.3	383	3.2	736	6.1	109.7	98.0	83.5	687	73.12	106.4	103.04	303	39.2	129	147.54
European	695	4917	7.1	1902	2.7	3015	4.3	506.4	102.1	67.9	2363	267.7	113.3	81.74	694	95.4	137.4	133.64
Total	1768	18,152	10.3	5586	3.2	12,566	7.1	1902.3	104.8	89.7	7547	906.15	120	124.04	1217	244.7	201	329

*No. Ind., number of individuals; Avg., average; S.D., standard deviation.*

### Genetic Differentiation Among Populations

Our analysis showed genetic clustering of populations corresponding to geographic regions: Eastern-Central Asia, Western Asia, Africa, and Europe (Figures 1B,C and Supplementary Figure 2B). In general, these clusters are consistent with the phylogenetic trees on the basis of CNVR frequencies (Supplementary Figure 2) and the Admixture analysis (Supplementary Figure 3). In the PCA analysis, the first coordinate PC1 separated European breeds from the other breeds, and PC2 reflected the genetic difference between Romanov and Texel sheep (Supplementary Figure 4). Of note, the results showed that Eastern-Central Asia, Western Asia, and Africa groups were clustered together and separated from European group, which indicated significant genetic differentiation among populations of different geographic origins.

### Distribution of CNVs and CNVRs Among Populations

**FIGURE 4:**
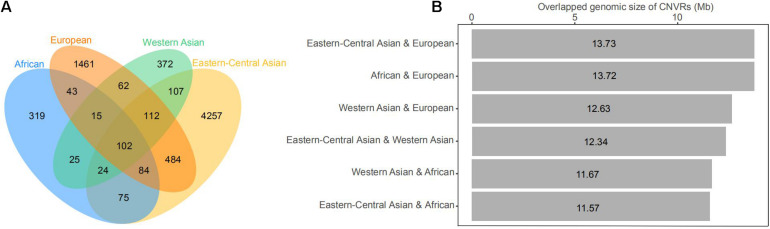
**(A)** Venn diagram of the number of copy number variations (CNVs) shared by geographic groups of domestic sheep, including African, Eastern-Central Asian, European, and Western Asian populations. **(B)** Histogram of genomic coverage by CNV regions (CNVRs) shared by two geographic groups of domestic sheep populations.

The CNVs shared by multiple groups can be considered as common variants (Figure 4 and Supplementary Tables 8,9). A large proportion of CNVRs (11.6–13.6 Mb) were shared across continents. Of the 1217 CNVRs, 709 (CNVR1 to CNVR709) contain at least two CNV events, with a cumulative length of 196 Mb and accounting for 8% of the sheep genome (Supplementary Table 10). CNVR1 and CNVR2 on chromosome 10 (Supplementary Table 10) have the highest frequencies, which are similar among the four geographic groups. Seven CNVRs have differential geographic distributions with a variance of > 0.02 across groups: CNVR3, CNVR20, and CNVR24 have an exceptional overrepresentation in West Asia and Africa, while CNVR6, CNVR7, CNVR12, and CNVR15 are overrepresented in Eastern Central Asia. In the clustering heatmap analysis, we selected the CNVRs whose variances ranked the top 5% of the 709 CNVRs (i.e., CNV events ≥ 2). The clustering matrices estimated from frequencies of the CNV events clearly differentiated the populations according to their geographic origins.

In total, 860 and 519 genes were annotated within the 1217 and 709 CNVRs, respectively (Supplementary Table 11). KEGG pathways analysis of the selective genes detected eight significantly (adjusted *P* < 0.05) enriched KEGG pathways (Supplementary Table 12). These include circadian entrainment (oas04713, *P* = 0.00006), phosphatidylinositol signaling system (oas04070, *P* = 0.000454223), oxytocin signaling (oas04921, *P* = 0.00064), glutamatergic synapse (oas04724, *P* = 0.012), inositol phosphate metabolism (oas00562, *P* = 0.0218), estrogen signaling (oas04915, *P* = 0.027), toxoplasmosis (oas05145, *P* = 0.00068), inflammatory bowel disease (oas05321, *P* = 0.0026), and intestinal immune network for IgA production (oas04672, *P* = 0.05). The significantly overrepresented pathways (adjusted *P* < 0.05) highlighted the main functional categories, such as energy metabolism and endocrine regulation, nervous system development and function, immune and inflammatory, and reproduction.

Of the 709 CNVRs with >1 recorded events, 107 CNVRs overlapped with 53 QTLs (Supplementary Table 13). Of the 107 CNVRs, 98 overlapped with QTLs for the production traits such as average daily gain, body weight, tail fat deposition, litter size, wool, and meat and milk production. In addition, 18 with QTLs related to immunoglobulin A level, Maedi-Visna virus susceptibility, ovine pulmonary adenocarcinoma susceptibility, and salmonella abortusovis susceptibility, suggesting that these CNVs might be informative for immunological functions.

### Comparison With Previous Sheep CNV Studies

To compare the identified CNVs in our study with those in previously published studies (Fontanesi et al., 2011; Hou et al., 2015), all CNVRs coordinates from original assemblies were migrated from Oar_v3.1 to Oar_v4.0 using the UCSC liftOver program^7^. The liftOver was carried out at normal thresholds (≥0.95 and ≥0.5), which are the minimum ratio of bases that must remap values. The CNVRs shared between this and previous studies were obtained using the BEDOPS v2.4.39 (Neph et al., 2012) (Supplementary Table 14).

### GWAS Between CNVs and Phenotypic Traits

**FIGURE 5:**
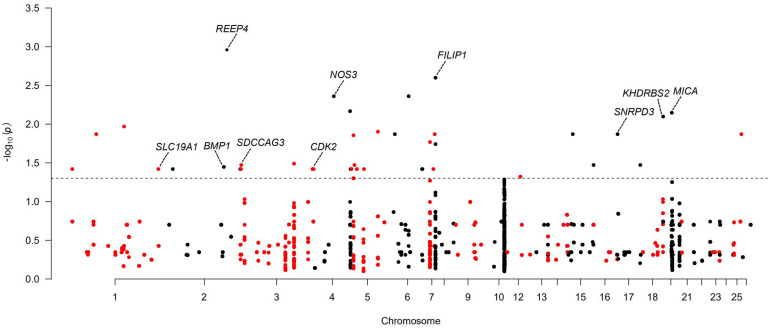
Manhattan plots of genome-wide association study (GWAS) of large litter size for Wadi, Hu, Finnsheep, Icelandic, and Romanov and smaller litter size for Texel. The threshold of adjusted *P* < 0.05 is indicated by a dotted line.

For the polyceraty, tail fat deposition, and nipple number phenotypes, we detected significant (adjusted *P* < 0.05) associations in four, eight, and two CNVRs, respectively (Supplementary Figures 5A–C and Supplementary Tables 15–17). These overlap with a set of novel functional genes. Thirty-six CNVRs are significantly associated with litter size (Figure 5 and Supplementary Table 18). Ten of these overlap with genes involved in reproduction and fertilization (CNVR5 and CNVR334 in Wadi; CNVR11, CNVR21, and CNVR100 in Hu; CNVR82, CNVR632, and CNVR334 in Icelandic sheep; CNVR234 in Finnsheep; CNVR47 in Romanov; CNVR504 in Texel). Six CNVRs (i.e., CNVR11, CNVR28, CNVR44, CNVR49, CNVR57, and CNVR66) are associated with ear size in Duolang sheep (Supplementary Figure 5D and Supplementary Table 19).

We compared the CNVRs identified here based on SNP BeadChip with those detected based on high-depth whole-genome sequences for the specific traits in one earlier study (Li et al., 2020a). We found that four CNVRs linked to litter size (three CNVRs) and tail type (one CNVR) here overlapped the CNVRs identified in an earlier investigation based on high-depth whole-genome sequences (Li et al., 2020a) (Supplementary Table 20).

## Discussion

In this study, we constructed a high-resolution sheep CNV map based on high-density SNP array genotypes and a worldwide panel of sheep populations. We observed considerable variations in the density of CNVs within the genome and of their frequencies across breeds from different geographic origins. In the genetic differentiation among populations, we observed similar genetic divisions as those based on Illumina 50K SNPs (Kijas et al., 2012; Zhao et al., 2017). We revealed a set of novel CNV-overlapping genes associated with local adaptation and phenotypic traits. The use of high-density set of genome-wide SNPs and strict filtering criteria has advanced the reliability and resolution in calling CNVs. Together with our large sample size, this has led to a detected CNVs coverage of approximately 10% of the sheep genome, which is higher than that found in previous sheep CNV studies (∼2.27–6.9%; Liu et al., 2013; Yang et al., 2018; Di Gerlando et al., 2019). As expected, African populations clustered together with the Western Asian populations, while Eastern-Central Asian populations were grouped together with European populations (Figure 2B), which was congruent with the worldwide pattern based on CNVs from a lower density of SNPs (Yang et al., 2018). Obviously, there is a higher number of loss events (*n* = 918) than the gain (*n* = 197) or both gain and loss (*n* = 102). This is agreement with previous findings (Mei et al., 2019; Prunier et al., 2019; Wang et al., 2019). For CNVs based on genome-wide SNP panels, it may be reasoned that during development of the panel SNPs, too many individuals of multicopy by gain events are deselected, while SNPs that by loss events have a low frequency of zero alleles are tolerated. Notably, most of the individual CNVs detected by qPCR experiments agreed well with the predicted positive samples identified by the PennCNV program.

Interestingly, KEGG pathways analyses of the genes within the 709 CNVRs highlighted their main physiological functions of energy metabolism, nervous system, immunity mechanism, and reproduction. Consequently, the CNVs are likely to modulate several types of relevant phenotypic traits and be involved in natural and artificial selection. Of the 1217 CNVRs, CNVR1 on chromosome 10 has 1497 events, showing by far the highest frequency in Eastern-Central Asia, followed by Europe, Western Asia, and Africa. It has a size of more than 1 Mb. Due to the incomplete genomic map of sheep, no annotated genes affecting polyceraty were located in this genomic region. Nevertheless, this region remained a strong candidate genomic region that was relevant to the polyceraty in sheep. In contrast, CNVR24 with a length of 85 kb has high frequencies only in Western Asia (69%) and Africa (23%). A similar but less contrasting pattern is shown by CNVR3 and CNVR20. Four other CNVRs show a geographic variance > 0.2 (CNVR6, CNVR7, CNVR12, and CNVR15; Supplementary Table 10), and they are overrepresented in Eastern-Central Asia. These observations suggested an involvement of CNVs in generic and environmental adaptation.

In the CNV-trait GWAS, CNVR93 (heterozygous loss) was found to be associated with polyceraty in Sishui Fur sheep with four horns. It has a frequency of 0.02841 in case group of four horns but not found in the control group of two horns. From the 32 genes overlapping with CNVR93 (Supplementary Table 15), we propose *SBNO2* as a novel candidate gene in CNVR93 for the polycerate phenotype. *SBNO2* has been found to be critical for osteoclast fusion and bone formation (El Kasmi et al., 2007; Okamoto et al., 2017).

For the tail weight in small- and large-tailed Han sheep, we found an association of CNVR16 (heterozygous gain), which overlaps with 13 genes (Supplementary Table 16). One of these genes, *PPP1R11*, encodes phosphatase 1 regulatory (inhibitor) subunit 11, which may be involved in Hippo signaling in apoptosis and cell proliferation, and has been linked to VLDL particle concentration in plasma (Kettunen et al., 2012; Piórkowska et al., 2018). Another candidate gene, *GABBR1*, overlaps with CNVR16 and encodes a receptor for gamma-aminobutyric acid (*GABA*) via the cAMP signaling pathway and regulates fatty acid β-oxidation and degradation. It has been reported to be associated with fat depot-selective adipose tissue macrophage infiltration in obesity (Hwang et al., 2019). Thus, our results suggest that *PPP1R11* and *GABBR1* are alternative candidate genes for the fat deposition in the tail of sheep.

For the supernumerary nipple phenotype in Wadi sheep, we observed a GWAS signal of CNVR14 (heterozygous loss), which overlaps with 17 genes (Supplementary Table 17) in the sheep with two (normal) nipples. Of the genes, *AKT1* is a gene of the Akt family and encodes protein controlling cellular metabolism, survival, and growth. It has been found to be associated with the regulation of regulating mammary tumor metastasis (Carpten et al., 2007). Thus, together with a previous SNP-based study in the same sheep (Peng et al., 2017), we propose *AKT1* as a candidate gene for supernumerary nipple phenotype in sheep, which functions via the regulation of cell proliferation.

In the five populations with relatively larger average litter size (Wadi, Hu, Icelandic, Finnsheep and Romanov) and one population with smaller average litter size (Texel), we detected several candidate genes associated with the variation in litter size (Supplementary Table 18). In Wadi sheep, we found GWAS signals for CNVR5 and CNVR334 (heterozygous gains), both overlapping with two functional genes. *NOS3* encodes endothelial nitric oxide synthase (eNOS), and its deficiency can lead to late menarche, reduced ovulation rates, fewer deliveries, and earlier onset of menopause in mouse (Hefler et al., 2002). Filamin A interacting protein 1 (*FILIP1*) plays a key role in ovarian dysfunction and female infertility by regulating DNA methylation (Zama and Uzumcu, 2013). In Hu sheep, we detected signals of CNVR11 (59 genes), CNVR21 (31 genes), and CNVR100 (1 gene), overlapping with the pregnancy-related genes *SNRPD3*, *SDCCAG3*, and *KHDRBS2*, respectively. *SNRPD3* has been shown to participate in early embryonic development in the Finnish Large White. Serologically defined colon cancer antigen-3 (*SDCCAG3*) plays a key role in spermatocytes and spermatids by regulating the completion of cytokinesis through its interaction with the protein tyrosine phosphatase *PTPN13* and the Arf GTPase-activating protein *GIT1* (Sakagami et al., 2016). *KHDRBS2* encodes an RNA-binding protein, which is tyrosine phosphorylated by Src during mitosis and is involved in mediating uterine endometrial stroma progenitor development (Saatcioglu et al., 2019). In Icelandic sheep, the three candidate genes *NOS3* (one of the two genes overlapping with CNVR334) (heterozygous gain), *BMP1* (one of the five genes overlapping with CNVR632) (heterozygous gain), and *SLC19A1* (one of two genes overlapping with CNVR82) (heterozygous gain) play important roles in the development of oocyte and embryogenesis. Interestingly, *NOS3* was also found in Wadi sheep. *BMP1* (bone morphogenetic protein) encodes a zinc-dependent metalloproteinase that belongs to the astacin family and contributes to maintain high levels of active transforming growth factor beta 1 (TGF-β1) in tissues by promoting the degradation of two TGF-β antagonists in the folliculogenesis of mammalian (Lei et al., 2016). Solute Carrier Family 19, member 1 (*SLC19A1*) has been involved in the recurrent miscarriages by mediating folate transport across cell membranes and maintaining intracellular concentrations of folate (Rah et al., 2012). In Finnsheep, *CDK2* overlaps with CNVR234. The gene encodes a member of a family of serine/threonine protein kinases, which is relevant to mammalian oocyte meiosis development through participating in cell cycle regulation (Dinarina et al., 2005). In Romanov sheep, major histocompatibility complex class I chain-related A (MICA) overlapping with CNVR47 (heterozygous gain) is critical for the development of tubal pathology through the binding of inhibitory receptors to its ligands (Mei et al., 2009). In Texel sheep, only CNVR632 gives a significant GWAS signal and overlaps with one annotated gene, receptor accessory protein 4 (*REEP4*). It encodes a membrane receptor accessory protein that may negatively influence fertility rates and has also been revealed to have a significant association with litter size in livestock (Ahn et al., 2014).

For the ear size in Duolang sheep, we identified GWAS signals for CNVR11, CNVR28, CNVR44, CNVR49, CNVR57, and CNVR66, overlapping with four functional genes (*CSRP1*, *WNT7B*, *HMX1*, and *FGFR3*). It was previously reported that these genes were involved in regulating the development of inner and outer ear. For example, *CSRP1* has been shown to participate in the development of inner ear hair cell in the mouse (Curtin et al., 2003). *HMX1* has also been revealed to have a significant association with dominantly inherited crop ears in Highland cattle (Koch et al., 2013).

Noteworthy, functional candidate genes identified from CNV-based association analyses reveal a set of novel functional candidate genes, which so far have not been detected in previous SNP-based studies. Thus, the integration of different types of genetic markers would be useful for future whole-genome association studies. We did not detect previously reported important CNVs for specific traits, for example, the 190 kb duplication in *ASIP* associated with coat color (Norris and Whan, 2008; Lye and Purugganan, 2019). The most probable explanation could be ascribed to the characteristics of BeadChip SNPs used here, rather than the high-coverage whole-genome sequences. Nevertheless, the *ASIP* gene is located approximately 566 kb upstream of the CNVR289 (chr13:62,425,794-62,485,091). In addition, we detected less CNVRs than earlier studies, which could be due to the following: (i) Instead of whole-genome sequences used in some earlier studies, SNP BeadChip was used for the CNV identification here, and (ii) stringent filtering criteria were applied in this study, and only samples with CNV call count <100 were included in this study. If the count of CNV calls by PennCNV exceeding 100 being suggestive of poor DNA quality, those samples were excluded in the analyses (Glessner et al., 2010; Li et al., 2020b).

Our analysis showed that most CNVRs in our study were overlapping with two previous studies with the same genotyping platform (Supplementary Table 14; Zhu et al., 2016; Ma et al., 2017). However, we did not find overlapping CNVRs for other traits between this and the earlier study, which could be ascribed to much smaller sample sizes for the other traits in both the studies. In addition, the reason could be that most of early studies are based on CNV detection using a lower density of SNPs. Instead, here, we implemented probe-based statistics for CNV occurrence in both cases–control design or population with quantitative trait using a high-density SNP BeadChip array, which should lead to more reliable associations. Finally, the difference in threshold value used to GWAS was also another potentially influential factor.

## Conclusion

To our knowledge, this is the most comprehensive CNV analysis of high-resolution CNV map in sheep, including a large set of samples from diverse populations across the world. We detected a set of CNV-associated genes involved in climatic adaptation and artificial selection. Our results provide valuable insights into the adaptive genome landscape and genetic basis of phenotypic traits in sheep. In addition, our findings also provide important resources of CNVs for future molecular breeding and improvement in sheep.

## Data Availability Statement

The original contributions presented in the study are included in the article/Supplementary Material, further inquiries can be directed to the corresponding author.

## Ethics Statement

Ethical review and approval was not required for the animal study because Only DNA was used in the study.

## Author Contributions

M-HL and F-HL conceived and designed the project. MS, LG, Y-HC, AE, and JL collected the samples. Y-XX extracted the DNA. JL provided help in Beadchip genotyping. HS-D, Y-XX, S-SX, XL, L-YL, Y-JL, D-FW, Z-HC, and JG analyzed the data. HS-D and S-SX wrote the manuscript with contributions from M-HL. All authors reviewed and approved the final manuscript.

## Conflict of Interest

The authors declare that the research was conducted in the absence of any commercial or financial relationships that could be construed as a potential conflict of interest.

## Footnotes

1 http://www.ncbi.nlm.nih.gov/genome?term=ovis%20aries

2 http://tree.bio.ed.ac.uk/software/figtree/

3 http://www.openbioinformatics.org/penncnv/

4 https://sourceforge.net/projects/parsecnv/files/ParseCNV20.tgz/

5 http://david.abcc.ncifcrf.gov/

6 https://www.animalgenome.org/cgi-bin/QTLdb/OA/index

7 http://genome.ucsc.edu/cgi-bin/hgLiftOver

## Supplementary Material

Supplementary Figure 1 Normalized ratio (NR) obtained by qPCR for the nine randomly selected CNVRs. The y-axis shows the NR values obtained by qPCR, and the x-axis shows the sample names in the different CNV regions. Samples with NRs of approximately two have the diploid normal copy numbers, samples with NRs of approximately have one loss-of-copy allele and samples with NRs of more than two have gain of copy number.

Supplementary Figure 2 Neighbor-joining clustering based on the Manhattan distances matrix computed from CNVs for each population.

Supplementary Figure 3 **(A)** ADMIXTURE analysis for *K* values 2 to 7 for the 67 domestic sheep populations. **(B)** Principal component analysis (PCA) of the domestic sheep populations. The circles of yellow, blue, green represent Eastern-Central Asian, Western Asian and African populations, respectively.

Supplementary Figure 4 Principal component analysis (PCA) of the domestic sheep populations for four geographic groups. Codes for the populations are detailed in supplementary table 1.

Supplementary Figure 5 Manhattan plots of GWAS of **(A)** polyceraty of Sishui Fur sheep; **(B)** tail weight of Han sheep; **(C)** supernumerary nipples of Wadi sheep; and **(D)** Ear size of Duolang sheep. The threshold of adjusted *P* < 0.05 is indicated by a dotted line.
