# Supplementary material for: Genome-Wide Detection of Copy Number Variations and Their Association With Distinct Phenotypes in the World’s Sheep
Source: Front Genet. 2021 May 20;12:670582. doi: 10.3389/fgene.2021.670582 (PMC8175073; doi:10.3389/fgene.2021.670582)
Supplement: Supplementary Figure 1 — Normalized ratio (NR) obtained by qPCR for the nine randomly selected CNVRs. The y-axis shows the NR values obtained by qPCR, and the x-axis shows the sample names in the different CNV regions. Samples with NRs of approximately two have the diploid normal copy numbers, samples with NRs of approximately have one loss-of-copy allele and samples with NRs of more than two have gain of copy number. [file Data_Sheet_1.zip › fgene.2021.670582 -Datasheet attached/Data Sheet 1.zip!/Supplementary Figures&Supplementary Tables/Supplementary Figure 1.PDF]

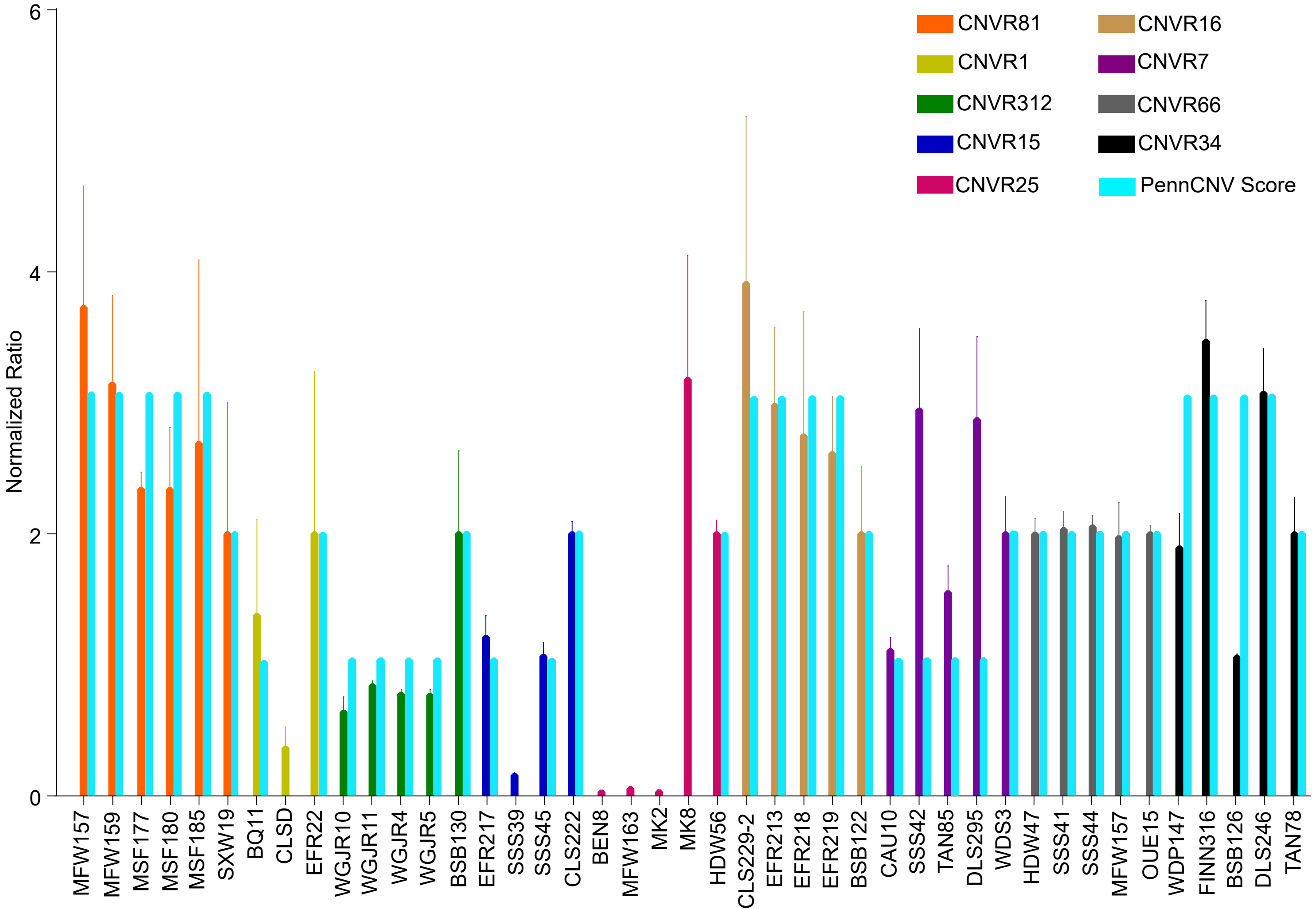

CNVR81
CNVR16
CNVR1
CNVR7
CNVR312
CNVR66
CNVR15
CNVR34
CNVR25
PennCNV Score
Normalized Ratio
0
2
4
6
MFW157
MFW159
MSF177
MSF180
MSF185
SXW19
BQ11
CLSD
EFR22
WGJR10
WGJR11
WGJR4
WGJR5
BSB130
EFR217
SSS39
SSS45
CLS222
BEN8
MFW163
MK2
MK8
HDW56
CLS229-2
EFR213
EFR218
EFR219
BSB122
CAU10
SSS42
TAN85
DLS295
WDS3
HDW47
SSS41
SSS44
MFW157
OUE15
WDP147
FINN316
BSB126
DLS246
TAN78
