# Supplementary material for: Genome-Wide Detection of Copy Number Variations and Their Association With Distinct Phenotypes in the World’s Sheep
Source: Front Genet. 2021 May 20;12:670582. doi: 10.3389/fgene.2021.670582 (PMC8175073; doi:10.3389/fgene.2021.670582)
Supplement: Supplementary Figure 1 — Normalized ratio (NR) obtained by qPCR for the nine randomly selected CNVRs. The y-axis shows the NR values obtained by qPCR, and the x-axis shows the sample names in the different CNV regions. Samples with NRs of approximately two have the diploid normal copy numbers, samples with NRs of approximately have one loss-of-copy allele and samples with NRs of more than two have gain of copy number. [file Data_Sheet_1.zip › fgene.2021.670582 -Datasheet attached/Data Sheet 1.zip!/Supplementary Figures&Supplementary Tables/Supplementary Figure 2.PDF]

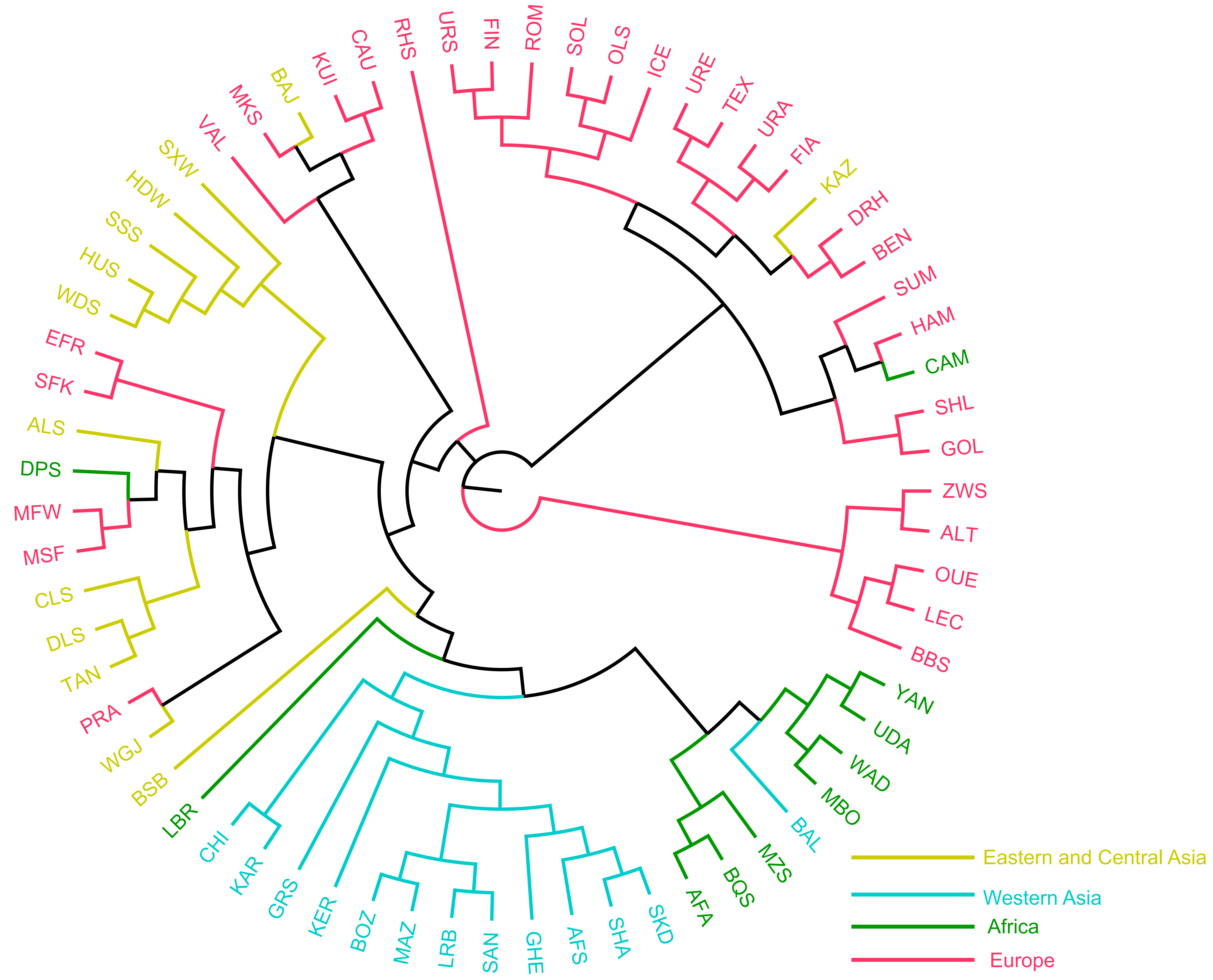
RHS
URS
FIN
ROM
SOL
OLS
ICE
URE
TEX
URA
FIA
KAZ
DRH
BEN
SUM
HAM
CAM
SHL
GOL
ZWS
ALT
OUE
LEC
BBS
YAN
UDA
WAD
MBO
BAL
MZS
BQS
AFA
SKD
SHA
AFS
GHE
SAN
LRB
MAZ
BOZ
KER
GRS
KAR
CHI
LBR
BSB
WGJ
PRA
TAN
DLS
CLS
MSF
MFW
DPS
ALS
SFK
EFR
WDS
HUS
SSS
HDW
SXW
VAL
MKS
BAJ
KUI
CAU
Eastern and Central Asia
Western Asia
Africa
Europe
