# Supplementary material for: Genome-Wide Detection of Copy Number Variations and Their Association With Distinct Phenotypes in the World’s Sheep
Source: Front Genet. 2021 May 20;12:670582. doi: 10.3389/fgene.2021.670582 (PMC8175073; doi:10.3389/fgene.2021.670582)
Supplement: Supplementary Figure 1 — Normalized ratio (NR) obtained by qPCR for the nine randomly selected CNVRs. The y-axis shows the NR values obtained by qPCR, and the x-axis shows the sample names in the different CNV regions. Samples with NRs of approximately two have the diploid normal copy numbers, samples with NRs of approximately have one loss-of-copy allele and samples with NRs of more than two have gain of copy number. [file Data_Sheet_1.zip › fgene.2021.670582 -Datasheet attached/Data Sheet 1.zip!/Supplementary Figures&Supplementary Tables/Supplementary Figure 3.PDF]

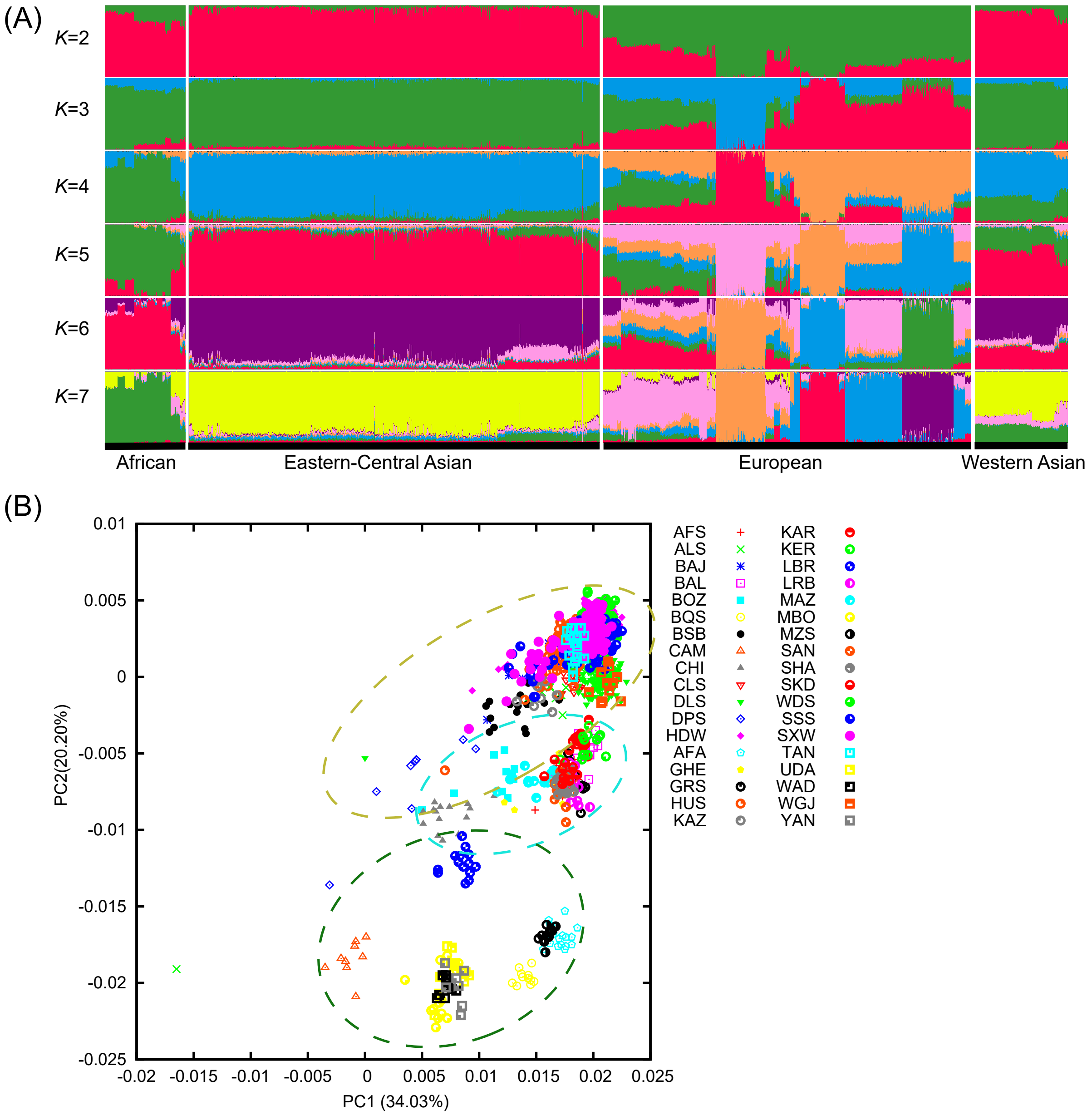

(A)
K=2
K=3
K=4
K=5
K=6
K=7
African
Eastern-Central Asian
European
Western Asian
(B)
PC1 (34.03%)
PC2(20.20%)
0.01
0.005
0
-0.005
-0.01
-0.015
-0.02
-0.025
-0.02
-0.015
-0.01
-0.005
0
0.005
0.01
0.015
0.02
0.025
AFS
ALS
BAJ
BAL
BOZ
BQS
BSB
CAM
CHI
CLS
DLS
DPS
HDW
AFA
GHE
GRS
HUS
KAZ
KAR
KER
LBR
LRB
MAZ
MBO
MZS
SAN
SHA
SKD
WDS
SSS
SXW
TAN
UDA
WAD
WGJ
YAN
