# Supplementary material for: Genome-Wide Detection of Copy Number Variations and Their Association With Distinct Phenotypes in the World’s Sheep
Source: Front Genet. 2021 May 20;12:670582. doi: 10.3389/fgene.2021.670582 (PMC8175073; doi:10.3389/fgene.2021.670582)
Supplement: Supplementary Figure 1 — Normalized ratio (NR) obtained by qPCR for the nine randomly selected CNVRs. The y-axis shows the NR values obtained by qPCR, and the x-axis shows the sample names in the different CNV regions. Samples with NRs of approximately two have the diploid normal copy numbers, samples with NRs of approximately have one loss-of-copy allele and samples with NRs of more than two have gain of copy number. [file Data_Sheet_1.zip › fgene.2021.670582 -Datasheet attached/Data Sheet 1.zip!/Supplementary Figures&Supplementary Tables/Supplementary Figure 4.PDF]

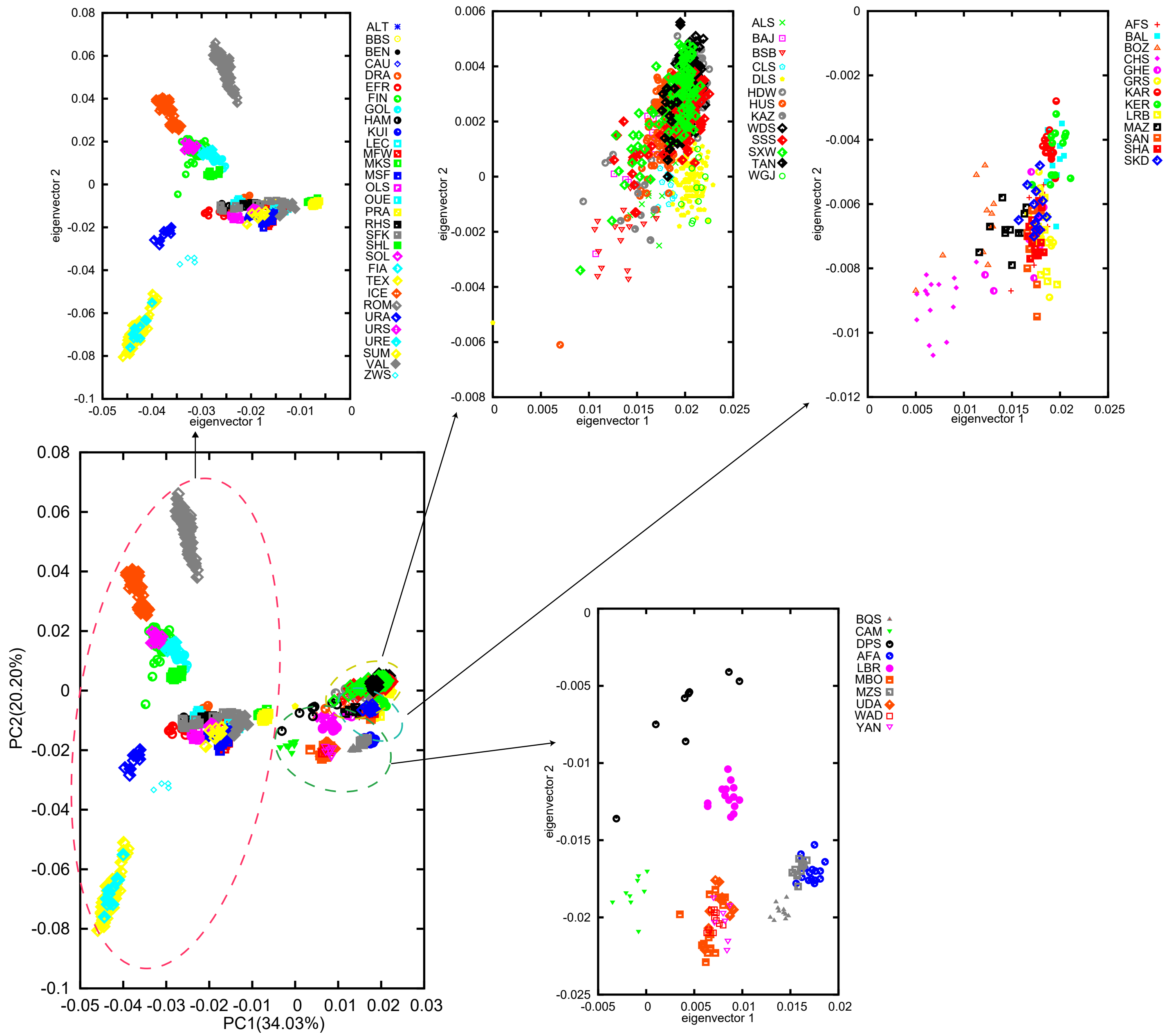

ALT
BBS
BEN
CAU
DRA
EFR
FIN
GOL
HAM
KUI
LEC
MFW
MKS
MSF
OLS
OUE
PRA
RHS
SFK
SHL
SOL
FIA
TEX
ICE
ROM
URA
URS
URE
SUM
VAL
ZWS
eigenvector 1
eigenvector 2
ALS
BAJ
BSB
CLS
DLS
HDW
HUS
KAZ
WDS
SSS
SXW
TAN
WGJ
AFS
BAL
BOZ
CHS
GHE
GRS
KAR
KER
LRB
MAZ
SAN
SHA
SKD
BQS
CAM
DPS
AFA
LBR
MBO
MZS
UDA
WAD
YAN
PC1(34.03%)
PC2(20.20%)
