# Supplementary material for: Genome-Wide Detection of Copy Number Variations and Their Association With Distinct Phenotypes in the World’s Sheep
Source: Front Genet. 2021 May 20;12:670582. doi: 10.3389/fgene.2021.670582 (PMC8175073; doi:10.3389/fgene.2021.670582)
Supplement: Supplementary Figure 1 — Normalized ratio (NR) obtained by qPCR for the nine randomly selected CNVRs. The y-axis shows the NR values obtained by qPCR, and the x-axis shows the sample names in the different CNV regions. Samples with NRs of approximately two have the diploid normal copy numbers, samples with NRs of approximately have one loss-of-copy allele and samples with NRs of more than two have gain of copy number. [file Data_Sheet_1.zip › fgene.2021.670582 -Datasheet attached/Data Sheet 1.zip!/Supplementary Figures&Supplementary Tables/Supplementary Figure 5.PDF]

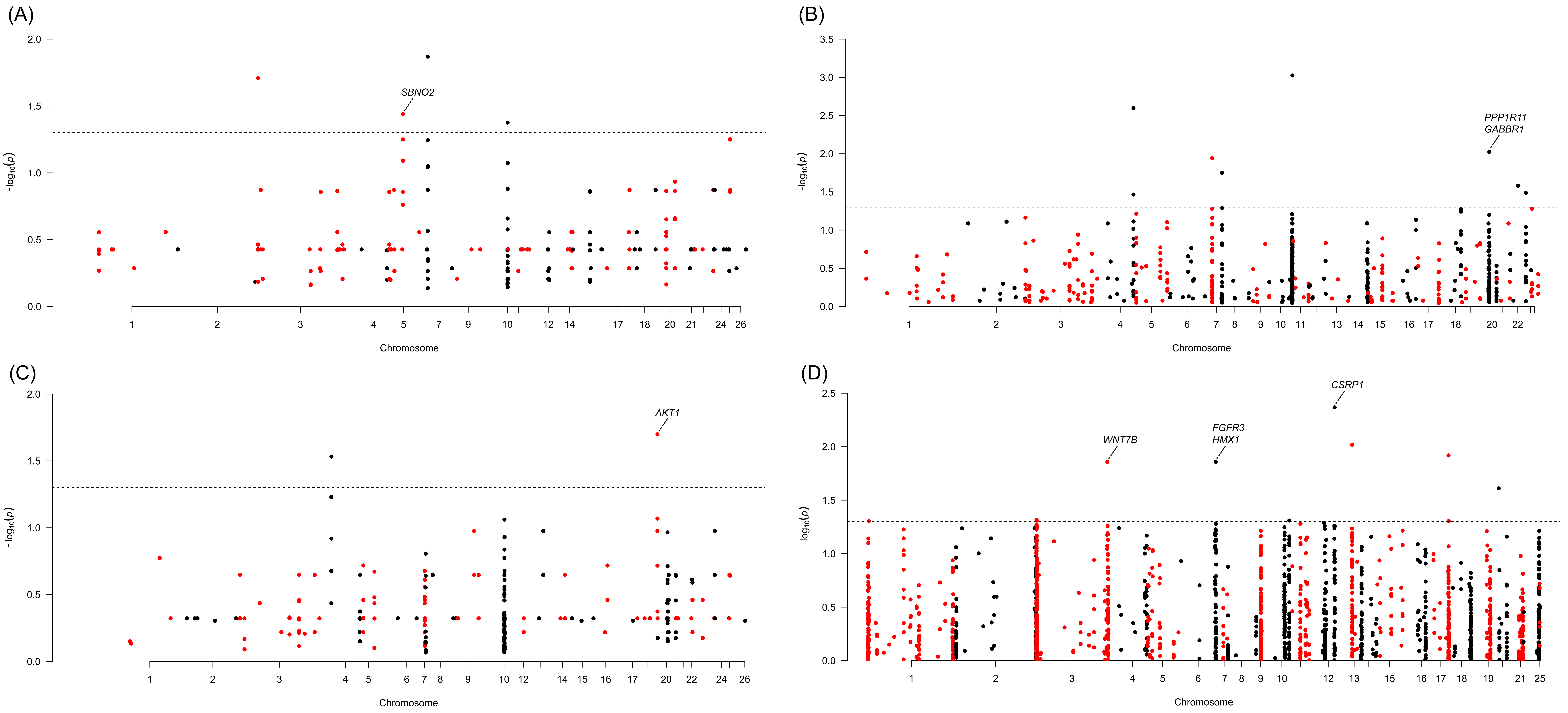
(A)
2.0
1.5
1.0
0.5
0.0
-log10(p)
SBNO2
1 2 3 4 5 7 9 10 12 14 17 18 20 21 24 26
Chromosome
(B)
3.5
3.0
2.5
2.0
1.5
1.0
0.5
0.0
-log10(p)
PPP1R11
GABBR1
1 2 3 4 5 6 7 8 9 10 11 13 14 15 16 17 18 20 22
Chromosome
(C)
2.0
1.5
1.0
0.5
0.0
-log10(p)
AKT1
1 2 3 4 5 6 7 8 9 10 12 14 15 16 17 20 22 24 26
Chromosome
(D)
2.5
2.0
1.5
1.0
0.5
0.0
log10(p)
CSRP1
WNT7B
FGFR3
HMX1
1 2 3 4 5 6 7 8 9 10 12 13 15 16 17 18 19 21 25
Chromosome
