# Supplementary material for: Genome-Wide Detection of Copy Number Variations and Their Association With Distinct Phenotypes in the World’s Sheep
Source: Front Genet. 2021 May 20;12:670582. doi: 10.3389/fgene.2021.670582 (PMC8175073; doi:10.3389/fgene.2021.670582)
Supplement: Supplementary Figure 1 — Normalized ratio (NR) obtained by qPCR for the nine randomly selected CNVRs. The y-axis shows the NR values obtained by qPCR, and the x-axis shows the sample names in the different CNV regions. Samples with NRs of approximately two have the diploid normal copy numbers, samples with NRs of approximately have one loss-of-copy allele and samples with NRs of more than two have gain of copy number. [file Data_Sheet_1.zip › fgene.2021.670582 -Datasheet attached/Data Sheet 2.zip!/Additional files/Data Sheet 7.PDF]

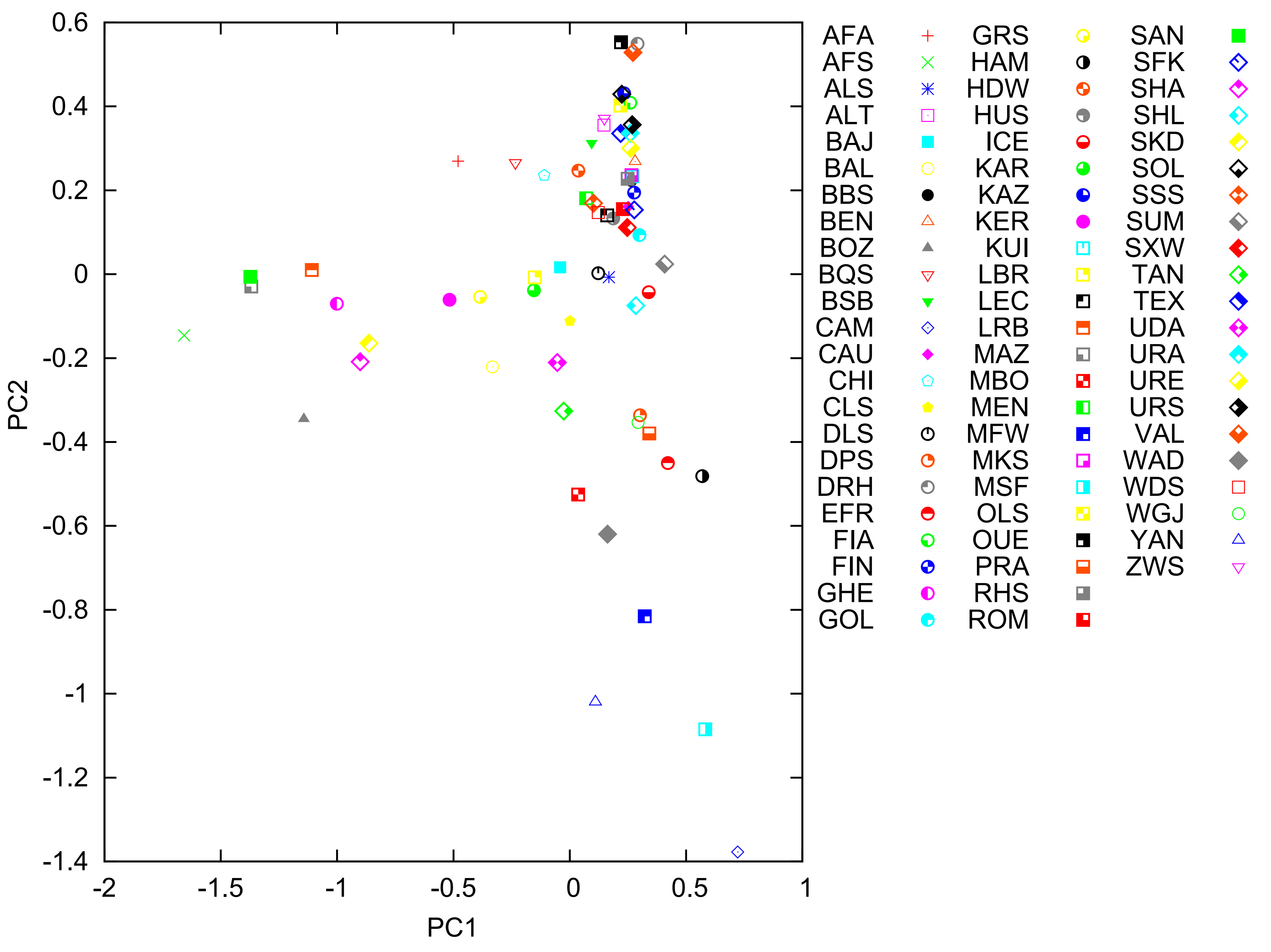

0.6
0.4
0.2
0
-0.2
-0.4
-0.6
-0.8
-1
-1.2
-1.4
-2
-1.5
-1
-0.5
0
0.5
1
PC1
PC2
AFA
AFS
ALS
ALT
BAJ
BAL
BBS
BEN
BOZ
BQS
BSB
CAM
CAU
CHI
CLS
DLS
DPS
DRH
EFR
FIA
FIN
GHE
GOL
GRS
HAM
HDW
HUS
ICE
KAR
KAZ
KER
KUI
LBR
LEC
LRB
MAZ
MBO
MEN
MFW
MKS
MSF
OLS
OUE
PRA
RHS
ROM
SAN
SFK
SHA
SHL
SKD
SOL
SSS
SUM
SXW
TAN
TEX
UDA
URA
URE
URS
VAL
WAD
WDS
WGJ
YAN
ZWS
