# Supplementary material for: Genome-Wide Detection of Copy Number Variations and Their Association With Distinct Phenotypes in the World’s Sheep
Source: Front Genet. 2021 May 20;12:670582. doi: 10.3389/fgene.2021.670582 (PMC8175073; doi:10.3389/fgene.2021.670582)
Supplement: Supplementary Figure 1 — Normalized ratio (NR) obtained by qPCR for the nine randomly selected CNVRs. The y-axis shows the NR values obtained by qPCR, and the x-axis shows the sample names in the different CNV regions. Samples with NRs of approximately two have the diploid normal copy numbers, samples with NRs of approximately have one loss-of-copy allele and samples with NRs of more than two have gain of copy number. [file Data_Sheet_1.zip › fgene.2021.670582 -Datasheet attached/Data Sheet 2.zip!/Additional files/Data Sheet 8.PDF]

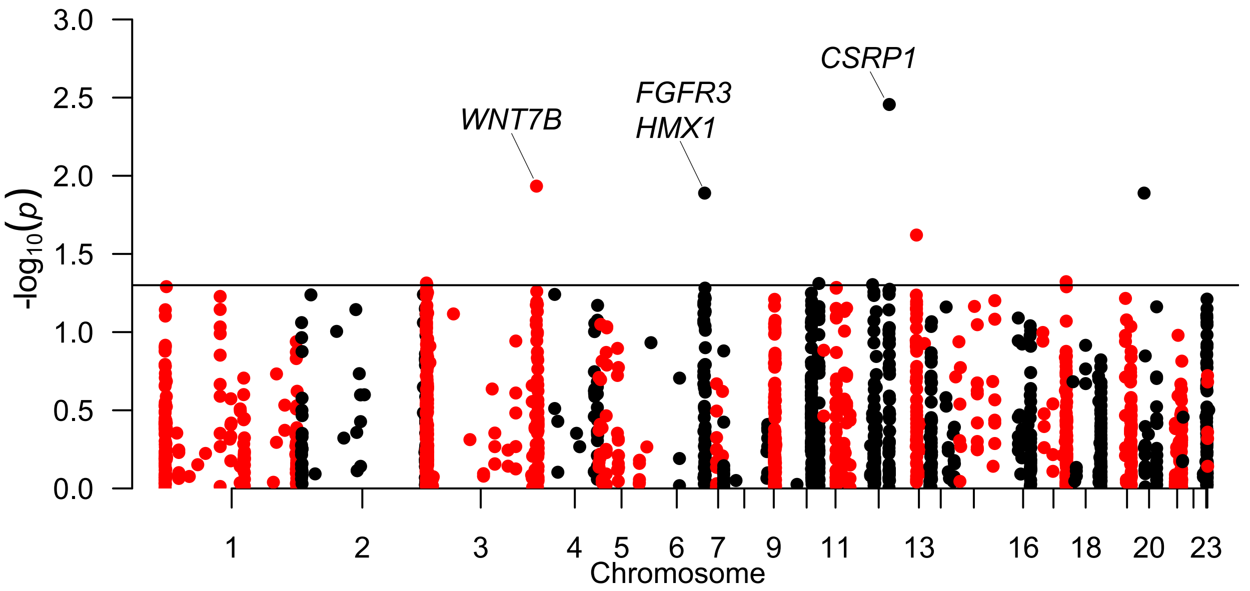

3.0
2.5
2.0
1.5
1.0
0.5
0.0
$-\log_{10}(p)$
WNT7B
FGFR3
HMX1
CSRP1
1
2
3
4
5
6
7
9
11
13
16
18
20
23
Chromosome
