# Supplementary material for: Genome-Wide Detection of Copy Number Variations and Their Association With Distinct Phenotypes in the World’s Sheep
Source: Front Genet. 2021 May 20;12:670582. doi: 10.3389/fgene.2021.670582 (PMC8175073; doi:10.3389/fgene.2021.670582)
Supplement: Supplementary Figure 1 — Normalized ratio (NR) obtained by qPCR for the nine randomly selected CNVRs. The y-axis shows the NR values obtained by qPCR, and the x-axis shows the sample names in the different CNV regions. Samples with NRs of approximately two have the diploid normal copy numbers, samples with NRs of approximately have one loss-of-copy allele and samples with NRs of more than two have gain of copy number. [file Data_Sheet_1.zip › fgene.2021.670582 -Datasheet attached/Data Sheet 2.zip!/Additional files/Table 3.DOCX]

We used the programs ParseCNV (https://sourceforge.net/projects/parsecnv/files/ ParseCNV20.tgz/) and PLINK v1.09 in the association analyses, applying the linear mixed model for tail weight and ear size. First, we used the program ParseCNV, which calculated probe-based statistics for CNV occurrence in both the linear mixed model on the basis of the CNV calls using the code “*--includePed argument*”. Next, we calculated the association between CNVs and phenotypic traits. We applied the ParseCNV script “*InsertPlinkPvalues.pl*” to merge adjacent SNP-based CNV occurrence p-values into CNVRs. Program ParseCNV was run with 10,000 permutations using the code “*--permuteP <10,000>*”. After the multiple testing correction, a nominal significance threshold of max(T) adjusted *P* < 0.05 was used to select CNVRs for pathway analysis. Associations between CNVs and continuous traits such as ear size and tail weight were analyzed according the following model:

$y=Xb+Za+e$

where y is a vector of phenotypes; *X* is the design matrix for the fixed effects; b is a vector of the fixed effects of overall mean, sex of animals (male, female), and probes for CNV occurrence ; a is a vector of the animal random effects; *Z* is the design matrix for the animal random effects; e is a vector of the residuals; a∼N(0, *σ_g_*^2^G), e∼N(0, *σ_e_*^2^I*n*); n is number of the animals, I is an identity matrix, *σ_g_*^2^ and *σ_e_*^2^ are the marker and residual variance components, respectively. The empirical correlation matrix (genomic relationship matrix, G) was estimated using the dense SNPs data.
